# Disparities in Depressive Symptoms and Antidepressant Treatment by Gender and Race/Ethnicity among People Living with HIV in the United States

**DOI:** 10.1371/journal.pone.0160738

**Published:** 2016-08-11

**Authors:** Angela M. Bengtson, Brian W. Pence, Heidi M. Crane, Katerina Christopoulos, Rob J. Fredericksen, Bradley N. Gaynes, Amy Heine, W. Christopher Mathews, Richard Moore, Sonia Napravnik, Steven Safren, Michael J. Mugavero

**Affiliations:** 1 Department of Epidemiology, University of North Carolina at Chapel Hill, Chapel Hill, North Carolina, United States of America; 2 Department of Medicine, School of Medicine, University of Washington, Seattle, Washington, United States of America; 3 HIV/AIDS Division, San Francisco General Hospital, University of California San Francisco, San Francisco, California, United States of America; 4 Department of Psychiatry, School of Medicine, University of North Carolina at Chapel Hill, Chapel Hill, North Carolina, United States of America; 5 Division of Infectious Diseases, Department of Medicine, School of Medicine, University of North Carolina at Chapel Hill, Chapel Hill, North Carolina, United States of America; 6 Department of Medicine, School of Medicine, University of California San Diego, San Diego, California, United States of America; 7 Department of Medicine, School of Medicine, Johns Hopkins University, Baltimore, Maryland, United States of America; 8 Department of Psychology, University of Miami, Miami, Florida, United States of America; 9 Department of Medicine and UAB Center for AIDS Research, University of Alabama at Birmingham, Birmingham, Alabama, United States of America; British Columbia Centre for Excellence in HIV/AIDS, CANADA

## Abstract

**Objective:**

To describe disparities along the depression treatment cascade, from indication for antidepressant treatment to effective treatment, in HIV-infected individuals by gender and race/ethnicity.

**Methods:**

The Center for AIDS Research (CFAR) Network of Integrated Clinical Systems (CNICS) cohort includes 31,000 HIV-infected adults in routine clinical care at 8 sites. Individuals were included in the analysis if they had a depressive symptoms measure within one month of establishing HIV care at a CNICS site. Depressive symptoms were measured using the validated Patient Health Questionnaire-9 (PHQ-9). Indication for antidepressant treatment was defined as PHQ-9 ≥ 10 or a current antidepressant prescription. Antidepressant treatment was defined as a current antidepressant prescription. Evidence-based antidepressant treatment was considered treatment changes based on a person’s most recent PHQ-9, in accordance with clinical guidelines. We calculated the cumulative probability of moving through the depression treatment cascade within 24 months of entering CNICS HIV care. We used multivariable Cox proportional hazards models to estimate associations between gender, race/ethnicity, and a range of depression outcomes.

**Results:**

In our cohort of HIV-infected adults in routine care, 47% had an indication for antidepressant treatment. Significant drop-offs along the depression treatment cascade were seen for the entire study sample. However, important disparities existed. Women were more likely to have an indication for antidepressant treatment (HR 1.54; 95% CI 1.34, 1.78), receive antidepressant treatment (HR 2.03; 95% CI 1.53, 2.69) and receive evidence-based antidepressant treatment (HR 1.67; 95% CI 1.03, 2.74), even after accounting for race/ethnicity. Black non-Hispanics (HR 0.47, 95% CI 0.35, 0.65), Hispanics (HR 0.63, 95% CI 0.44, 0.89) and other race/ethnicities (HR 0.35, 95% CI 0.17, 0.73) were less likely to initiate antidepressant treatment, compared to white non-Hispanics.

**Conclusions:**

In our cohort of HIV-infected adults depressive symptoms were common. Important disparities in the prevalence of depressive symptoms and receipt of antidepressant treatment existed by gender and race/ethnicity.

## Introduction

Depression affects an estimated 30% of HIV-infected adults, making it the most commonly reported mental health condition among adults in HIV care.[1, 2]. For people living with HIV (PLHIV), depression negatively affects quality of life,[3–5] and is consistently associated with reduced antiretroviral (ART) adherence, [6–11] viral suppression,[12–14] and higher mortality rates.[12, 14] Despite the high prevalence of depression and its association with poor outcomes among HIV-infected adults, depression remains widely unrecognized, untreated, or undertreated in HIV clinical care.[15, 16]

The “depression treatment cascade”, similar to the HIV treatment cascade, estimates the proportion of HIV-infected adults with depression whose condition is recognized clinically, treated, and remits. Estimates of the depression treatment cascade suggest that less than half of all cases of depression are recognized clinically. Of cases recognized clinically, only half receive any mental health treatment, and fewer than half of those treated receive guideline-concordant treatment.[17] Although these large gaps in depression identification and treatment among HIV-infected patients are well documented, little is known about gender and racial/ethnic disparities that may exist in this cascade.

In the general population, disparities in the prevalence, diagnosis, and treatment of depression are complex. A higher lifetime prevalence of depression has been reported among white non-Hispanics (18%), compared to black non-Hispanics (11%) and Hispanics (14%).[18] However, black Americans are also more likely to be undiagnosed[19] and to experience more severe or persistent depression.[20, 21] Hispanics and black Americans are also less likely to receive antidepressant medication,[22–24] including treatment that conforms to current clinical guidelines [21, 25] or to be referred for counseling.[26, 27] Disparities in depression care by gender also exist. Compared to men, women are significantly more likely be diagnosed with depression [28] and to utilize mental health services.[29, 30] Differences in the assessment of depression between studies, from a diagnosis of major depressive disorder using the Diagnostic and Statistical Manual of Mental Disorders [31] to validated scales which screen for depressive symptoms, such as the Patient Health Questionaire-9, [32] further complicates our understanding of disparities in depression prevalence and treatment.

Less is known about disparities in depression care that may exist in HIV-infected populations by gender or race/ethnicity. As HIV transitions from an acute to a chronic medical condition, high-quality mental health services to ensure the long-term health of HIV-infected adults are increasingly important. In the United States, HIV disproportionately affects men who have sex with men (MSM) and women in the southern United States–two groups also at high risk of depression.[28, 33–36] Given the high burden of depression among HIV-infected adults and disparities by gender and race/ethnicity in the general population, understanding disparities in depression care among HIV-infected adults is essential to optimize mental health and clinical outcomes.

The goal of the present analysis is to describe disparities along the depression treatment cascade by gender and race/ethnicity in a large cohort of HIV-infected adults in the United States. We report on differences by gender and race/ethnicity in the cumulative probability of successfully moving through the depression treatment cascade, from indication for antidepressant treatment to effective treatment, and estimate the association between gender, race/ethnicity and a range of depression-related outcomes.

## Materials and Methods

Data for the present analysis come from the Center for AIDS Research (CFAR) Network of Integrated Clinical Systems (CNICS) cohort. The CNICS cohort includes over 31,000 HIV-infected adults in routine HIV clinical care at 8 sites in the United States.[37] CNICS collects detailed information on demographic characteristics, ART, antidepressant medications, HIV/AIDS clinical events, co‐morbid conditions, HIV‐related laboratory values and vital status on patients who consent to participate. Starting between 2005 and 2011, patients in CNICS also began completing self-administered socio‐behavioral questionnaires, or Patient‐Reported Outcomes (PROs), on touch-screen tablets as part of routine care visits. HIV-infected patients complete PROs approximately every 4–6 months, although this varies based on clinical care follow-up. Written informed consent was obtained from all study participants and documented at each CNICS site. Ethical approval for the use of routinely collected clinical data was provided by the institutional review board at the University of North Carolina at Chapel Hill.

### Study Population

The present analysis includes HIV-infected individuals in CNICS who had a PRO measure, which includes a depression measure, within one month of establishing HIV care at a CNICS site. For this study, we selected two CNICS sites that routinely initiate the PRO assessment at the initial visit (Site A and Site B). Site A is in the southern United States and Site B is in the western United States. Several CNICS sites typically wait until the 2^nd^ or later follow-up visit for the initial PRO assessment due to competing clinical demands for a patient who is new to HIV care, such as introducing the patient to the case manager, addressing insurance and fiscal concerns, etc. Furthermore, the two sites selected provide substantial clinical and racially/ethnic diversity including Hispanics, African-Americans, and women. Participants were followed from entry into care at a CNICS site for up to 24 months or until a depression-related outcome (defined below), administrative censoring in September or October, 2014 (depending on site), loss to follow-up (LTFU; defined as 12 months after a participant’s last HIV visit) or death, whichever date came first.

### Measures

Each PRO includes an assessment of depressive symptoms using the Patient Health Questionnaire-9 (PHQ-9).[32] The PHQ-9 has been widely validated, including among HIV-infected populations.[38, 39] PROs also include several other validated measures for mental health and substance use, including: panic disorder (Patient Health Questionnaire-5),[40] high risk alcohol use (The Alcohol Use Disorders Identification Test (AUDIT), defined as ≥ 4 for males and ≥ 3 for females),[41] and use of illicit drugs including cocaine/crack, heroin/opiates, crystal/amphetamine use, but excluding marijuana (The Alcohol, Smoking and Substance Involvement Screening Test (ASSIST)).[42, 43] Information on counseling-based depression treatment is not available in CNICS.

**Fig 1:**
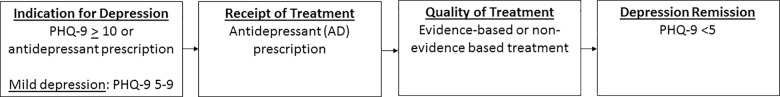
The depression treatment cascade for HIV-infected adults.

For the present analysis, we focused on disparities in indication, receipt and quality of antidepressant treatment among HIV-infected adults (Fig 1; Table 1). We defined indication for antidepressant treatment as a PHQ-9 total score ≥ 10 (88% sensitivity and specificity to indicate a major depressive episode)[32] or prescription of an antidepressant. Receipt of antidepressant treatment was defined as prescription of an antidepressant medication. Quality of antidepressant treatment was considered to be evidence-based or not evidence-based. Evidence-based antidepressant treatment was defined as adjusting the antidepressant prescription based on follow-up depressive severity measures in a manner consistent with evidence-based guidelines. [44, 45] Specifically, for patients on antidepressant treatment, if a follow-up PHQ-9 score was ≥10 (indicating persistent depressive symptoms) and either the antidepressant dose was increased or a switch or augmentation of the antidepressant regimen was prescribed, this was considered active evidence-based treatment. Whereas if no medication adjustment occurred, this was considered not to be evidence-based treatment. If the follow-up PHQ-9 score was <10, then both changes to and maintenance of the antidepressant regimen were considered to be (passive) evidence-based. Secondary outcomes considered were mild depressive symptoms (PHQ-9 between 5 and 9), and depression remission (PHQ-9 <5).[46] We examined depression-related outcomes stratified by gender (female or male) and race/ethnicity (white, non-Hispanic; black, non-Hispanic, Hispanic, or other).

**Table 1:** Depression-related outcomes and definitions.

Outcome	Definition
Indication for antidepressant treatment	PHQ-9 ≥ 10 or current antidepressant medication prescription
Antidepressant treatment	Current antidepressant medication prescription
Evidence-based antidepressant treatment	Among those on an antidepressant: Dose increase or medication switch following a PHQ-9 score ≥ 10; dose increase, medication switch, or no change following a PHQ-9 score <10.
Active evidence-based antidepressant treatment	Among those on an antidepressant: Dose increase or medication switch following a PHQ-9 score ≥ 10.
Mild depressive symptoms	PHQ-9 5–9
Depression remission	PHQ-9 <5 among patients taking an antidepressant

### Data Analysis

To understand disparities in depression treatment and response across the depression treatment cascade, we conducted three analyses–each examining a different aspect of the depression treatment experience. First, to understand the likelihood of a person sequentially moving through the depression treatment cascade, we looked at each individual’s antidepressant treatment experience over 24 months and calculated the cumulative probability of ever having an indication for antidepressant treatment, ever initiating antidepressant treatment, ever receiving evidence-based antidepressant treatment, and ever receiving active evidence-based antidepressant treatment. The cumulative probability of each event was defined as the probability of experiencing an event, multiplied by the probability of experiencing each previous event on the depression treatment cascade.

Second, to understand the depression treatment cascade at the clinic population level, we categorized the first 24 months of each individual’s person-time in CNICS HIV care by its depressive severity (not depressed, mild depressive symptoms, depressed) and antidepressant status (on or not on antidepressants) and stratified by gender and race/ethnicity. Antidepressant status was determined using patient medication records for each person-month under observation. PHQ-9 measurements were considered valid (i.e. carried forward) for up to 6 months. Person-time was excluded if it had been more than 6 months since the most recent PHQ-9 measure.

Finally, we used multivariable Cox proportional hazards models to estimate associations between gender, race/ethnicity, and the following depression treatment cascade outcomes over 24 months: first indication for antidepressant treatment, first receipt of antidepressant medication, and first receipt of evidence-based antidepressant treatment. The origin was considered entry into care at the CNICS site for all multivariable analyses and all models included site and calendar time, modeled using restricted cubic splines. Additional covariates, such as CD4, viral load, substance use and other mental health disorders, were not included because they likely represent mediators, rather than confounders, of the relationship between race/ethnicity, gender and depression outcomes. We developed inverse probability of censoring weights to account for differential LTFU by baseline demographic (age) and clinical factors (depression, anxiety and viral load suppression) that were associated with LTFU.[47] The likelihood of receiving active evidence-based depression treatment and achieving depression remission were not evaluated in multivariable analyses due to limited sample size. All analyses were conducted using Stata 13 (StataCorp: College Station, TX).

## Results

Overall 1,390 persons met our inclusion criteria of having a PHQ-9 measure within one month of entering HIV care (Table 2) and contributed a total of 17,138 person-months of follow-up time. There were 24 deaths (2%) during the follow-up period. The proportion of individuals who were lost to follow-up ranged from 12% when an outcome of indication for antidepressant treatment was considered, to 19% when an outcome of evidence-based antidepressant treatment was considered. The majority of participants were from Site B (81%). Participants were largely male (89%), white non-Hispanic (49%) and MSM (68%). Overall, a larger proportion of men were white non-Hispanic (51%) compared to women (35%), with the largest proportion of women being black non-Hispanic (38%). Over half of participants (63%) had an unsuppressed viral load at entry into CNICS HIV care, and the median CD4 count was 427 cells/mm^3^. Substance abuse was common; 16% of participants reported current drug use, 39% past drug use and 33% high risk alcohol use. Compared to women, men were more likely to report past drug use (40% versus 33%) and high risk alcohol use (34% versus 24%).

**Table 2:** Sociodemographic, clinical and mental health characteristics at entry into care of 1,390 HIV-infected adults in CNICS.

	N (%) or Median (IQR)
*Demographic and clinical characteristics*^1^	
**Age**	41 (32, 49)
**Gender**	
Male	1,234 (88.8)
Female	156 (11.2)
**Race/ethnicity**	
White, non-Hispanic	680 (49.1)
Black, non-Hispanic	290 (20.9)
Hispanic	328 (23.7)
Other	87 (6.3)
**Risk group**	
IDU	146 (10.6)
MSM (no IDU)	942 (68.3)
Heterosexual (no MSM or IDU)	237 (17.2)
Other	55 (4.0)
**Site**	
Site A	270 (19.4)
Site B	1,120 (80.6)
**Viral load at enrollment**	
Undetectable, < 50 copies/mL	497 (36.9)
Detectable, ≥ 50 copies/mL	849 (63.1)
**CD4 count, cells/mm3**	427 (230, 639)
≤ 200	301 (21.9)
201–500	532 (38.7)
>500	543 (39.5)
**ART use**	
Efavirenz containing	176 (12.7)
Non-efavirenz containing	345 (24.8)
Not on ART	869 (62.5)
**Panic disorder**	
No panic	921 (67.1)
Some panic symptoms	246 (17.9)
Panic disorder	205 (14.9)
**Drug use (excluding marijuana use)**	
No use	544 (45.3)
Current use	191 (15.9)
Past use	465 (38.8)
**High risk alcohol use**	
Yes	444 (32.8)
No	910 (67.2)
*Mental health characteristics over 24 months of follow-up*	
PHQ-9 indicating depressive symptoms (≥10)	543 (39.1)
Indication for antidepressant treatment (A)	659 (47.4)
Of A: Initiated antidepressant treatment (B)	290 (44.0)
Of B: Evidence-based antidepressant treatment (C)	116 (40.0)
Of C: Active evidence-based antidepressant treatment	21 (18.1)
Depression remission (at any time over 24 months)	109 (20.1)

1 Missing data: race/ethnicity 0.5%, risk group 0.7%, viral load 3.2%, CD4 count 1.0%, panic disorder 1.3%, drug use 13.7%, alcohol use 2.6%.

The prevalence of mental health issues in our cohort was high. At entry into CNICS care, one third of participants had some indication of panic disorder (either some panic symptoms or panic disorder). Panic disorder was more common among women than men (27% versus 14%). Over 24 months of follow-up, nearly 40% of participants ever had a PHQ-9 ≥ 10 indicating depressive symptoms and almost half (47%) had either a PHQ-9 indicating depressive symptoms or were on an antidepressant at some point (Table 2; S1 Table). Women were more likely than men to have a PHQ-9 indicating probable depression (54% versus 37%).

**Fig 2:**
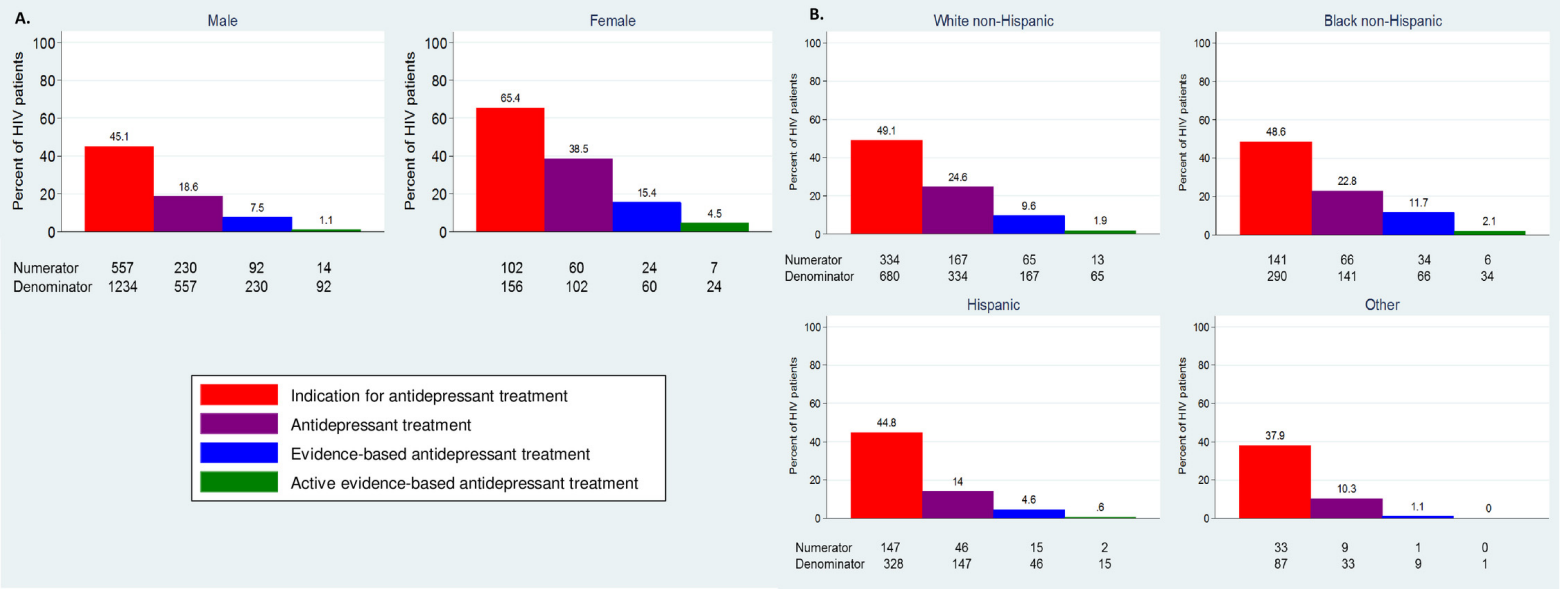
The cumulative probability of moving through the depression treatment cascade, by (A) gender and (B) race/ethnicity. The cumulative probability of experiencing each depression-related event is defined as the probability of experiencing an event, multiplied by the probability of experiencing each previous event on the depression treatment cascade.

There was a substantial decline in the proportion of patients who progressed to each subsequent category along the depression treatment cascade for all genders and race/ethnicities (Fig 2A). However, important disparities existed. Over 65% of women had an indication for antidepressant treatment within 24 months of entering CNICS HIV care, while only 45% of men did. Women also had a higher cumulative probability of receiving antidepressant treatment within 24 months of entering care (39% versus 19% for men). Overall, 40% of individuals who initiated antidepressant treatment received evidence-based antidepressant treatment (Table 2). However, the vast majority of evidence-based antidepressant treatment was passive (the patient had a low PHQ-9 score and therefore no clinician action was indicated); only a small proportion was active evidence-based (dose increase or medication switch following a high PHQ-9). The cumulative probability of having an indication for antidepressant treatment, receiving antidepressant treatment and for that treatment to be evidence-based was low for both genders (15% for women and 8% for men). The cumulative probability of active evidence-based treatment was even lower (5% for women and 1% for men). Among persons who had a PHQ-9 ≥ 10 (n = 543), 20% experienced depression remission at some point during follow-up, regardless of antidepressant treatment.

When the depression treatment cascade was stratified by race/ethnicity, 38–49% of persons in each group had an indication for antidepressant treatment (Fig 2B). However, the cumulative probability of receiving antidepressant treatment (e.g. the probability of having an indication for antidepressant treatment and subsequently receiving treatment) was higher among white non-Hispanics (25%) and black non-Hispanics (23%), compared to Hispanics (14%) and persons of other race/ethnicity (10%). The cumulative probability of evidence-based antidepressant treatment was also low across all racial/ethnic groups, but again was higher for white non-Hispanics (10%) and black non-Hispanics (12%), compared to Hispanics (5%) and persons of other race/ethnicity (1%). The cumulative probability of receiving active evidence-based antidepressant treatment was very low in all racial/ethnic groups (0–2%).

**Fig 3:**
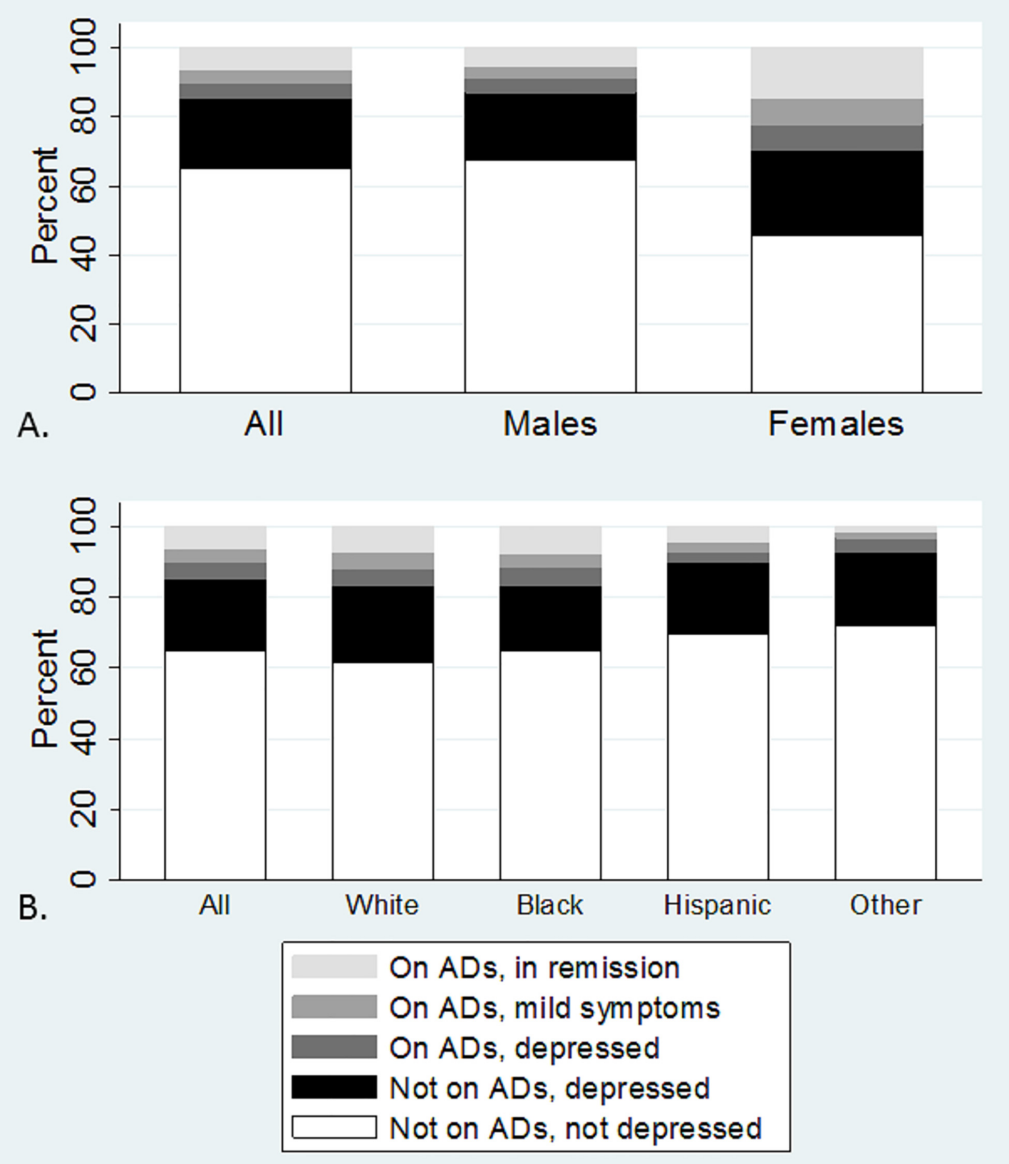
Antidepressant (AD) use and depressive severity, stratified by person-time and (A) gender and (B) race/ethnicity.

When person-time was stratified by depressive severity and antidepressant use, women spent considerably more time with depressive symptoms or on antidepressants than men (approximately 50% versus 30%; Fig 3). However, women also spent more time in remission while on antidepressants (approximately 15%), compared to men (approximately 5%). When the proportion of individuals who experienced depression remission (on antidepressant treatment with a PHQ-9 score < 5) was considered, similar results were seen among men (12%) and women (13%).

When person-time stratified by depressive severity and antidepressant use was considered by race/ethnicity, the amount of person-time spent on antidepressants was similar between white non-Hispanics and black non-Hispanics (approximately 15% of person-time for both groups; Fig 3). Hispanics and those of other race/ethnicity experienced slightly lower proportions of person-time on antidepressants (approximately 10% and 7%, respectively). Across gender and racial/ethnic groups, a sizeable proportion of person-time (approximately 20%) was spent with depressive symptoms but not on antidepressant treatment.

In multivariable analyses accounting for both gender and race/ethnicity, the largest disparities in having an indication for antidepressant treatment and receiving treatment persisted by gender. Compared to men, women were more likely to have an indication for depression treatment (HR 1.54, 95% CI 1.34, 1.78); Table 3). Black non-Hispanics (HR 0.47, 95% CI 0.35, 0.65), Hispanics (HR 0.63, 95% CI 0.44, 0.89) and persons of other race/ethnicity (HR 0.35, 95% CI 0.17, 0.73) were less likely to initiate antidepressant treatment, compared to white non-Hispanics. Women were also more likely to initiate antidepressant treatment (HR 2.03, 95% CI 1.53, 2.69), and to receive evidence-based depression treatment (HR 1.67, 95% CI 1.03, 2.74). Compared to white non-Hispanics, all other racial/ethnic groups were also less likely to receive evidence-based depression treatment, however estimates were imprecise due to limited sample size. Associations between gender and race/ethnicity with active evidence-based antidepressant treatment were not evaluated due to small sample size.

**Table 3:** Multivariable results for the associations between gender and race/ethnicity with depression-related events.^1^

Characteristic	Indication for Antidepressant Treatment	Initiate Antidepressant Treatment	Evidence-based Antidepressant Treatment
	HR (95% CI)	HR (95% CI)	HR (95% CI)
Male	1.00	1.00	1.00
Female	1.54 (1.34, 1.78)	2.03 (1.53, 2.69)	1.67 (1.03, 2.74)
White, non-Hispanic	1.00	1.00	1.00
Black, non-Hispanic	0.86 (0.72, 1.03)	0.47 (0.35, 0.65)	0.48 (0.30, 0.79)
Hispanic	0.84 (0.70, 0.99)	0.63 (0.44, 0.89)	0.67 (0.37, 1.19)
Other	0.77 (0.56, 1.04)	0.35 (0.17, 0.73)	0.15 (0.02, 1.09)

1 All models adjusted for variables in the table, site, calendar time and inverse probability of censoring weights for LTFU.

## Discussion

In our cohort of HIV-infected adults in routine care, almost half of the population had an indication for antidepressant treatment. There was a substantial decline in the proportion of patients who proceeded to each subsequent category of the depression treatment cascade: from having an indication for antidepressant treatment, to starting antidepressant treatment, to receiving evidence-based antidepressant treatment or active evidence-based antidepressant treatment. Among those with depressive symptoms, the cumulative probability of receiving evidence-based antidepressant treatment remained ≤ 15% regardless of gender or race/ethnicity.

Our analysis revealed important disparities between men and women along the depression treatment cascade for HIV-infected adults. Women were more likely to have an indication for antidepressant treatment, to start antidepressant treatment and to receive evidence-based antidepressant treatment compared to men, even after accounting for race/ethnicity. Since only 11% of the study population were women, data were too sparse to examine gender-by-race interactions. However, similar rates of lifetime depression have been reported among both white non-Hispanic and black non-Hispanic women elsewhere.[48] Efavirenz, an antiretroviral drug with known psychiatric side effects, could account for some of the gender disparity in HIV-infected populations.[49] However, in our cohort only 13% of participants were on an efavirenz containing ART regimen at baseline and of those, only 5% were women. A higher proportion of depression among women has also been reported in the general US population.[28] The increased likelihood of women receiving any antidepressant treatment or evidence-based antidepressant treatment may be related to the fact that women are more likely than men to seek mental health services.[50, 51] Future analyses should explore possible reasons, such as domestic violence, economic vulnerability or social support, for gender disparities in depressive symptoms among PLWH.

Several large surveys of adults in the general US population have found a higher prevalence of depression among white non-Hispanics compared to black non-Hispanics or Hispanics.[18, 20, 52] In unadjusted analyses, the probability of having an indication for antidepressant treatment was similar across all race/ethic groups (ranging from 38%-49%). However, after adjusting for gender in the multivariable analysis, white non-Hispanics were more likely to have an indication for antidepressant treatment compared to black non-Hispanics and Hispanics, although confidence intervals included the null value of 1.00. Women in our cohort were more likely to be black non-Hispanic (38% versus 19% for men) and depressed (65% versus 45% for men), which may explain why accounting for gender resulted in a higher burden of depression on white non-Hispanics relative to other racial/ethnic groups.

In addition to having a higher prevalence of depression, white non-Hispanics in the general population also disproportionately are prescribed antidepressants and receive treatment in accordance with clinical guidelines.[21, 23, 24, 53–55] Our analysis demonstrated similar trends in a HIV-infected population, with white non-Hispanics being more likely to initiate antidepressant treatment and to receive evidence-based antidepressant treatment than all other race/ethnicities. The disparity in depression treatment for racial and ethnic minorities may be related to healthcare access and antidepressant prescribing patters of clinicians. Compared to white non-Hispanics, black non-Hispanics and Hispanics in the general population are more likely to be uninsured,[19, 24, 56] are less likely to receive an antidepressant prescription from a provider[55, 57] and are also less likely to fill an antidepressant prescription.[54] Many HIV-infected adults have access to HIV medical care through federal Ryan White HIV/AIDS program, however coverage for mental health services varies widely by state which may contribute to the disparity in initiating antidepressant treatment.[58] Non-medication-based depression interventions, such as counseling, have effectively reduced depression for non-white racial groups.[59–61] However information on counseling and psychotherapy was not available in our data.

Our analysis included participants from two sites in the southeastern and western United States. As a result, our study population was largely male, MSM and had a higher proportion of black non-Hispanics and Hispanics than HIV-infected adults in the US overall.[62] Our study results, therefore, are generalizable to similar populations of HIV-infected adults, but may be less generalizable to HIV-infected adults in the US overall. Further, participants in our analysis were receiving HIV care at large academic medical centers, which may have more resources to screen for and treat depression in HIV-infected populations. Additional work is needed to characterize the prevalence of depressive symptoms and disparities in depression treatment, with both antidepressant and counseling-based approaches, for HIV-infected adults receiving care at smaller, community-based clinics.

Our study has several important limitations. Depressive symptoms were defined in our cohort as a PHQ-9 score ≥10, which is a validated threshold for identifying probable depression but is not diagnostic. Further, some antidepressants have secondary indications, such as bupropion for smoking cessation or duloxetine for pain management and more severe mental illnesses, such as bipolar disorder, are sometimes misclassified as depressive symptoms. Thus there may be some measurement error in our estimate of the proportion of the study population having an indication for antidepressant treatment. Second, although the CNICS dataset contains a unique combination of repeated PHQ-9 measures and antidepressant medication changes, we did not have data on referral to counseling or to additional aspects of the clinical presentation that might legitimately prompt a clinician not to adjust antidepressant treatment even in the face of persistent depressive symptoms (for example, concern about side effects at a higher dose or evidence of patient non-adherence to the prescribed antidepressant dose). Therefore some of the clinical care classified as “not evidence-based” may have been clinically appropriate. Finally, our study draws on a large, geographically and racially diverse populations of HIV-infected adults. However, our analysis was limited to participants at sites where the PHQ-9 is routinely administered upon entry into CNICS care to ensure a valid measure of depressive symptoms. Information on whether patients entering CNICS care have a new HIV diagnosis or have been in care previously is not available in CNICS. Consequently, the high prevalence of depressive symptoms reported in our study may in part reflect participants dealing with a new diagnosis of HIV.

## Conclusions

Our analysis is among the first to characterize disparities in indication, receipt and quality of antidepressant treatment among HIV-infected adults in routine care. The precipitous drop-offs we observed along the depression treatment cascade highlight the need to improve access to high-quality depression care for all HIV-infected adults. Our results also confirm that many of the disparities in receiving high-quality depression care that exist by gender and race/ethnicity in the general population, persist among HIV-infected adults. In particular, HIV-infected women appear to be experiencing more depressive symptoms compared to men, and racial/ethnic minorities are less likely to initiate antidepressants and receive evidence-based treatment than white non-Hispanics. Given the high burden of depression among HIV-infected adults, these disparities are particularly concerning. Increasing awareness among mental health and HIV providers about disparities in depression treatment for HIV-infected adults may help to close the treatment gap. However, future research should also explore whether different types of depression treatment interventions (such as antidepressant treatment only or antidepressant treatment with counseling) are more efficacious for women or racial/ethnic minorities in order to reduce disparities along the depression treatment cascade.

## Supporting Information

S1 Table Medication type and dose information for 290 persons initiating antidepressant treatment over up to 2 years of follow-up. (DOCX)
