# Supplementary material for: Disparities in Depressive Symptoms and Antidepressant Treatment by Gender and Race/Ethnicity among People Living with HIV in the United States
Source: PLoS One. 2016 Aug 11;11(8):e0160738. doi: 10.1371/journal.pone.0160738 (PMC4981370; doi:10.1371/journal.pone.0160738)
Supplement: S1 Table — (DOCX) [file pone.0160738.s001.docx]

|  | **Proportion on medication*** | **Dose range (milligrams)** |
| --- | --- | --- |
| **SSRI** |  |  |
| Citalopram | 44.80% | 10-60 |
| Escitalopram | 11.10% | 5-20 |
| Fluoxetine | 8.00% | 10-60 |
| Paroxetine | 19.80% | 10-60 |
| Sertraline HCL | 11.10% | 25-300 |
|  |  |  |
| **SNRI** |  |  |
| Desvenlafaxine | 3.50% | 100-100 |
| Duloxetine | 8.30% | 20-90 |
| Fenlafaxine | 9.00% | 25-300 |
|  |  |  |
| **Second generation** |  |  |
| Martazapine | 6.30% | 7.5-45 |
| Bupropion | 14.90% | 75-450 |

**S1 Table**. Medication type and dose information for 290 persons initiating antidepressant treatment over up to 2 years of follow-up.

*Proportions will add up to >100% because persons could have been on more than one antidepressant medication over the follow-up period.
